# Supplementary material for: Usefulness of Protocolized Point-of-Care Ultrasonography for Patients with Acute Renal Colic Who Visited Emergency Department: A Randomized Controlled Study
Source: Medicina (Kaunas). 2019 Oct 28;55(11):717. doi: 10.3390/medicina55110717 (PMC6915595; doi:10.3390/medicina55110717)
Supplement: Supplementary file 1 [file medicina-55-00717-s001.pdf]

Supplementary material

Table S1. Acute kidney injury stage (KIDIGO, Critical care 2016).

AKI Stage: Serum Creatinine Criteria		
AKI stage	1	Increase of serum creatinine by ≥0.3 mg/dl (≥26.4 μmol/L)<br>or<br>increase to 1.5–1.9 times from baseline
	2	Increase of serum creatinine to 2.0–2.9 times from baseline
	3	Increase of serum creatinine ≥3.0 times from baseline<br>or<br>serum creatinine > = 4.0 mg/dl (≥354 μmol/L)<br>or<br>treatment with renal replacement treatment<br>or<br>in patients <18 years, decrease in estimated GFR to <35 ml/min per 1.73 m^2

If the patient did not visit OPD of urology or did not perform follow up laboratory creatinine test in the OPD, the patients contacted by phone for interview at 30 days. The questions by the phone interview with patients were as follows including uremic symptoms:

Table S2. Phone interview questions.

Interview Questions	Check
1. Have you been to other hospital?	
-What made you go to hospital?	
-Were you diagnosed with another disease?	
-Were you treated with extracorporeal shock wave lithotripsy?	
2. Have you had any symptoms such as anorexia, nausea, fatigue, muscle cramps since discharge?	
3. Have you had any symptoms such as urinary frequency, dysuria or urgency with fever or chilling sense since discharge?	

Table S3. Grade of hydronephrosis.

Hydronephrosis Grade (Point of Care Ultrasound, 1e)	
Mild	Enlargement of the renal calices with preservation of the renal papillae.
Moderate	Rounding of the renal calices, obliteration of renal papillae, and blunting of medullary pyramids
severe	Calyceal ballooning, complete obliteration of papillae and pyramids and cortical thinning.

Table S4. Information and test results of ultrasonography in the ultrasound group.

**Practitioner**		**Self-Competence of Ultrasonography**	
Attending staff	10 (14)		
Fellow	8 (11)	Low	56 (77)
Grade of residency 4	3 (4)	Moderate	17 (23)
3	17 (23)	High	0
2	35 (48)		
**Renal Ultrasonography Experience (cases)**		**Grade of Hydronephrosis**	
30–49	35 (48)	None	24 (33)
50–99	17 (23)	Mild	44 (60)
>100	21 (29)	Moderate	5 (7)
		Severe	0

Data are shown as n (%). total number = 73.
